# Supplementary figures and images for: Comparative Transcriptome Analysis Reveals a Potential Regulatory Network for Ogura Cytoplasmic Male Sterility in Cabbage (Brassica oleracea L.)
Source: Int J Mol Sci. 2023 Apr 4;24(7):6703. doi: 10.3390/ijms24076703 (PMC10094764; doi:10.3390/ijms24076703)

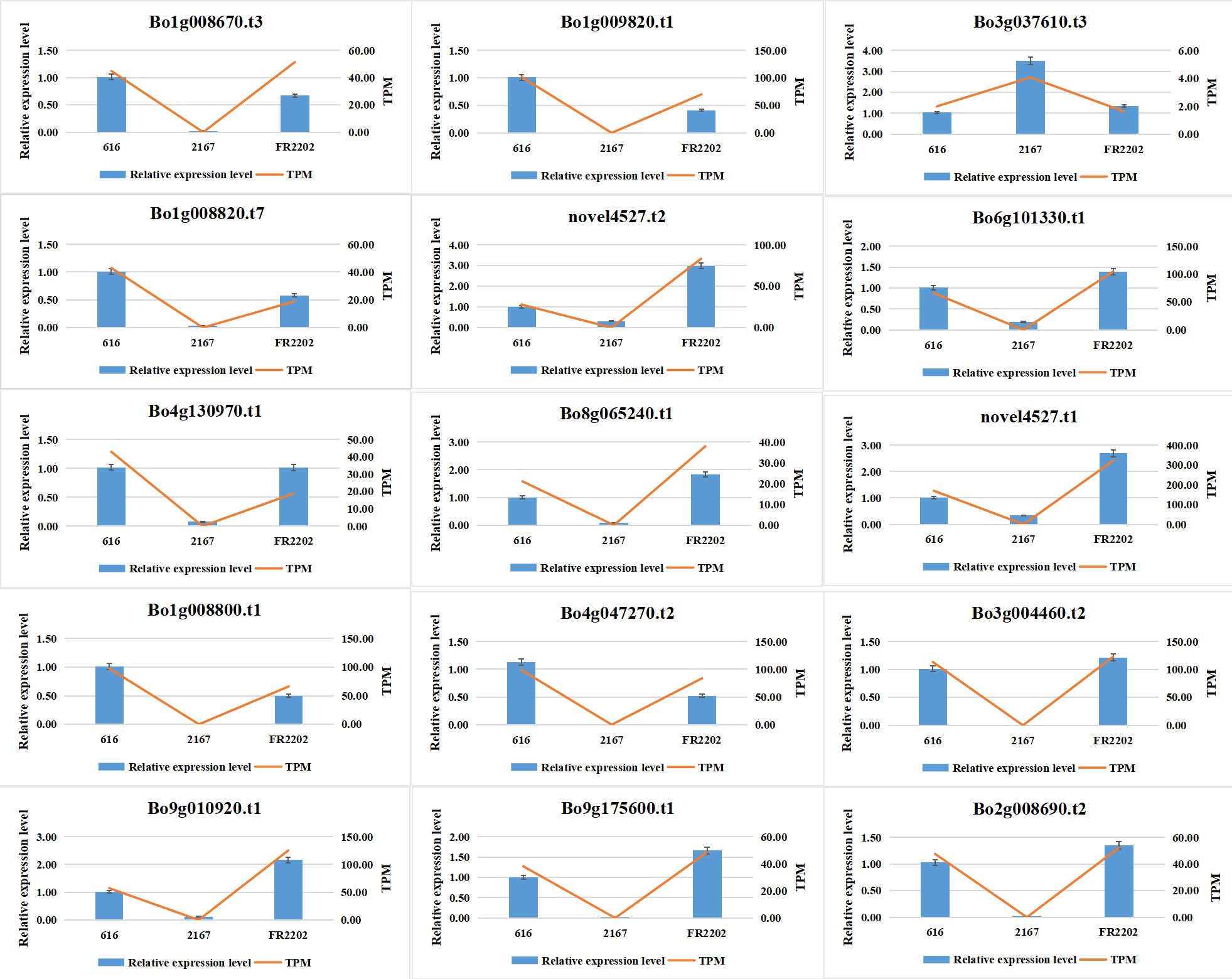

Supplement: Supplementary file 1 [file ijms-24-06703-s001.zip › Figure S1 Real-time quantitative PCR analysis.png]
